# An explainable multi-task deep learning framework for crash severity prediction using multi-source data

**DOI:** 10.1038/s41598-025-09226-1

**Published:** 2025-07-01

**Authors:** Yuanyuan Xiao, Zongtao Duan

**Affiliations:** 1 School of Information Engineering, Chang’ an University, Xi’an, 710064 China; 2 Department of Internet of Things and Network Engineering, Chang’ an University, Xi’an, 710064 China

**Keywords:** Crash severity prediction, Multi-task learning, Explainable AI (XAI), Multi-source traffic data, Risk factors, Engineering, Mathematics and computing

## Abstract

Traffic accidents pose significant global challenges, causing substantial injuries, fatalities, and economic losses. Current research predominantly focuses on single-prediction objectives (e.g., fatality prediction) while neglecting property damage assessments and critical interactions between prediction tasks. Although neural networks demonstrate superior predictive capabilities, their application in traffic safety analysis remains constrained by inherent limitations in causal interpretability, coupled with challenges posed by data imbalance, heterogeneity, and complexity in crash datasets. This study proposes an interpretable multi-task learning framework (Adv MT-DNN) that synergistically integrates an enhanced deep neural network with post-hoc explanation methods for comprehensive crash severity prediction. Our dual-focused approach addresses multiple prediction targets (including fatalities, severe injuries, and property damage). It provides granular insights into contributing factors through SHAP-based feature importance rankings and interaction analysis. Validated using four-year (2018–2021) multi-source traffic data from China, the framework demonstrates significant improvements in prediction accuracy compared to baselines. Nonparametric estimation of the top-8 critical factors (e.g., blood alcohol content, collision type, and accident occurrence period) confirms statistically significant associations with crash severity. The explicit interpretation mechanism bridges the critical gap between predictive performance and model interpretability in traffic safety analytics, providing engineering-relevant insights. This research establishes a robust methodological foundation for developing data-driven road safety policies and intelligent transportation systems, particularly in developing countries with complex traffic ecosystems.

## Introduction

Every year, traffic accidents result in many injuries, fatalities, and property damage, making serious threats to public safety and well-being worldwide. In 2019, 62,763 persons died in road accidents in China, with property damage amounting to 1.3 billion yuan^1,2^. To develop effective traffic safety policies that reduce injuries, deaths, and property losses, it is crucial to predict the severity of traffic accidents^3^.

Crash injury severity prediction models are used to forecast the severity of crashes and identify the factors influencing accident outcomes^4^. These models include discrete outcomes, data mining, and soft computing models^5,6^. Discrete outcome models, such as logit and probit models, calculate the probability of certain outcomes depending on particular attributes. The logit model, which utilizes a logistic function, is commonly used to analyze binary outcomes and can be extended to situations with multiple categories. Some studies have employed the ordered probit model to predict the degree of injuries in large-truck collisions, considering several variables such as collision characteristics, vehicle properties, and driver attributes^7,8^. Data mining techniques aim to discover patterns in large, complex datasets. Decision tree is a popular data mining approach that classifies data using a tree structure^9^. Researchers have developed decision tree ensemble models with information root node variation to predict fatal collisions involving novice drivers in urban locations^10^. Another widely used technique is the Bayesian network^4^. Studies have created driver and autonomous Bayesian networks to predict two injury severity outcomes: property damage and injury/fatality^11^.

In recent years, soft computing approaches, particularly neural networks, have been widely used for their ability to identify hidden patterns despite imprecision and uncertainty. These non-parametric models, consisting of interconnected artificial neurons, excel at handling datasets with outliers and missing values^12^. Some studies have utilized single-layer perceptron networks to classify injury severity, while others have employed backpropagation networks to predict the severity of injuries or fatalities^13^. A more advanced approach in traffic safety modeling is the use of deep neural networks (DNNs), which have demonstrated extraordinary performance. DNNs are particularly effective because they transform raw data into a latent space that simplifies prediction tasks. Despite their ability to capture intricate relationships with the data, their internal workings are difficult to interpret, leading them to be described as “black box” models. Nonetheless, their ability to provide accurate forecasts makes them valuable tools for remaining highly attractive tools for traffic accident severity prediction.

While neural networks are increasingly used to predict accidents, their results are not always straightforward. To address this challenge, sensitivity analysis (SA) has been widely used to understand how uncertainties in input features affect model outputs^14^. This approach applies to nearly all soft computing and data mining methods^15^. In the realm of model interpretability, several advanced methods have emerged to make predictions from complex models more understandable. One notable contribution in this area is Local Interpretable Model-Agnostic Explanation (LIME), which approximates the predictions of complex models by focusing on a specific data point. LIME provides insight into how input features influence the prediction by assigning weights based on the similarity between the instance and the model’s prediction, thereby making the model’s behavior more transparent^16^. Another powerful method is the Shapley Additive Explanation (SHAP), which is grounded in cooperative game theory. SHAP values offer a unified and consistent measure of feature importance by quantifying the contribution of each feature to the model’s output. This approach helps to explain how individual features interact to influence the predictions, offering a deeper understanding of the model’s decision-making process^17^.

Traditional statistical models, constrained by linear assumptions and dimensionality challenges (e.g., logistic regression’s inability to capture nonlinear interactions between road geometry and driver behavior), fail to address the complexity of multi-source traffic data^18^. While machine learning offers superior predictive power, its application remains fragmented. Existing studies predominantly rely on single-task models^19^, which leads to inconsistent risk interpretations across different severity types. For example, the factors influencing fatalities versus property damage are often analyzed in isolation, which obscures shared causal pathways. Though effective in static scenarios, current interpretability frameworks struggle in dynamic, multi-task environments. Explanations for correlated severity outcomes, such as injury severity and collision type, often contradict traffic engineering principles due to the lack of coordinated post-hoc analyses^20^. Moreover, despite advances in handling class imbalance through techniques like SMOTE, existing methods overlook cross-task feature rarity (e.g., rare weather-event interactions that simultaneously influence multiple severity dimensions), resulting in biased generalizability^21^. Crucially, the field lacks a unified approach that integrates multi-task learning with physics-informed interpretability, leaving practitioners to navigate trade-offs between predictive accuracy and actionable insights. These gaps are further amplified in heterogeneous contexts, such as China’s mixed traffic flows, where conventional feature selection methods inadequately capture localized risk patterns.

This study aims to build an interpretable multi-task learning framework with high performance and uncover the underlying mechanisms of various factors on the severity of traffic accidents by multi-sources data in China. The main contributions are outlined below: (1) Developing a multi-source fusion framework that systematically integrates five-dimensional determinants (driver behavior, vehicle characteristics, road, environmental conditions, and temporal) from authoritative governmental databases for crash severity modeling. (2) Establishing a hybrid interpretability paradigm combining the feature contribution and interaction effects of factors based on post-hoc interpretability and discovering some insights by incorporating non-parametric analysis to validate discovered patterns. (3) Mitigating the black-box challenge in machine learning models and overcoming the accuracy-interpretability trade-off in crash prediction systems especially when dealing with imbalanced and heterogeneous accident data.

## Data and methodology

**Fig. 1:**
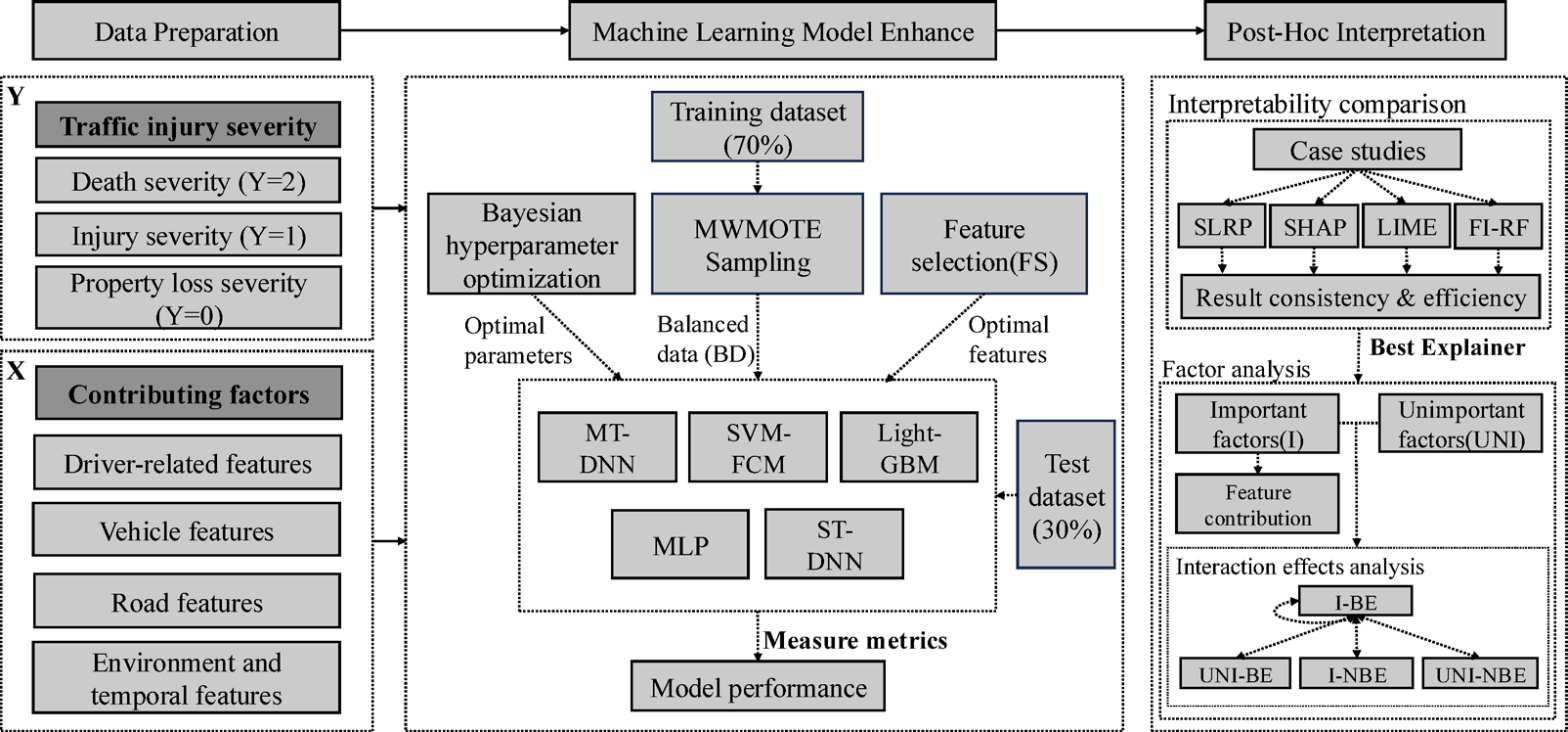
The research framework.

This study simultaneously considered multiple types of severity—fatalities, injuries, and property loss—along with four groups of attributes: driver-related characteristics, vehicle features, road features, and environmental and temporal factors. The subsequent sections will provide a detailed description based on this framework. The research framework is illustrated in Fig. 1.

### Data collection and preprocessing

The present study utilized data extracted from the Integrated Application Platform for Public Security and Traffic Management in Shaanxi Province, China, covering the period from August 2018 to August 2021. The multi-source data integrates various factors, including the environmental and temporal conditions of the crash, road characteristics, and driver and vehicle information. The severity of injuries is measured by the number of individuals harmed, while the severity of fatalities is reflected in the number of deaths resulting from the accident. Property loss is measured by the total damage value expressed in Chinese yuan. To this end, of the 9,957 traffic records, 4,276 (42.95%) are considered property loss crashes, 4,949 (49.70%) are considered injury (e.g., serious and slight) crashes, and 732 (7.34%) instances are fatal crashes, as shown in Table 1.

**Table 1:** Description of crash severity.

Severity	Category		Description	Max.	Min.	Median	Mean	Std.
Property loss (Y = 0)	Ordinal (42.95%)	7	0 1–99 100–999 1,000–9,999 10,000–99,999 100,000-999,999 1,000,000+	600,000	0	1,300	6,457	22,111
Injury (Y = 1)	Ordinal (49.70%)	9	0 1 2 3 4 5–10 11–15 16–20 20+	44	0	1	1.5	3.1
Fatal (Y = 2)	Ordinal (7.34%)	9	0 1 2 3 4 5–10 11–15 16–20 20+	5	0	0	0.1	0.4

Building on the analysis of previous studies and the 4 M theory, we extracted potential influential factors categorized into four groups: vehicle characteristics, driver attributes, roadway characteristics, and environmental variables. These aspects were identified based on research findings and the selection and organization of essential data, considering variables such as occurrence time, road and environmental characteristics, vehicle type, and accident features. Based on feature properties and feature selection (FS), the study processed variables as either continuous or categorical to ensure appropriate handling in the machine learning models. The data preprocessing stage includes cleaning the data, removing noise or irrelevant information, and binarizing and discretizing the data into a suitable format for training. By dividing continuous traffic data into discrete units, each unit can be treated as a sample and undergo feature extraction and encoding. A summary of independent variables is given in Table 2.

Most accident samples exhibit significant imbalance. Studies have shown that such sampling techniques can effectively address class imbalance. This study used the Majority Weighted Minority Oversampling Technique (MWMOTE) algorithm based on the Synthetic Minority Oversampling Technique (SMOTE)^22^. Unlike traditional oversampling methods, MWMOTE introduces a parameter *k* to control the minimum distance between minority class samples. (i) Determination of the weight of each minority class sample is calculated as ${w_i}=f\left( {{x_i}} \right)$, where ${w_i}$ represents the weight of the sample ${x_i}$. (ii) Based on the sample weights, the number of synthetic samples to be generated for each sample ${x_i}$ is determined as ${N_{new}}={N_{total}} \cdot {W_i}$, where ${N_{new}}$is the number of new samples generated for ${x_i}$ and ${N_{total}}$is the total number of synthetic samples to be generated, and (iii) for each selected minority class sample ${x_i}$, new synthetic samples ${x_{new}}$are generated by selecting its *k*-nearest neighbors ${x_j}$ and using the formula ${x_{new}}={x_i}+\delta ({x_j} - {x_i})$, $\delta$ is a randomly selected weight factor, typically in the range of [0,1].

**Table 2:** Independent variables.

Variable	Description	Freq. (%)
Gender (Gd)	1 = Male 2 = Female	7564 (76.00) 2389 (24.00)
Age (Ag)	1 = 18–25 2 = 26–45 3 = 46–65 4 = 65+	916 (9.78) 4537 (48.50) 3113 (33.24) 798 (8.52)
Driving experience (De)	1 = under 10 years 2 = 11–15 3 = 16–20 4 = 20+	3091 (64.24) 910 (18.91) 440 (9.14) 371 (7.71)
Alcohol content (Bac)	1 = Drunk driving 2 = Sober	3834 (38.51) 6123 (61.49)
Drug abuse (Da)	1 = Used 2 = Not used	1159 (11.64) 8798 (88.36)
Safety device usage (Sdu)	1 = No seatbelts 2 = Other	2459 (24.70) 7498 (75.30)
Educational level (Ed)	1 = Advanced education 2 = Higher education 3 = Secondary and Vocational education 4 = Other	2 (0.02) 274 (2.75) 615 (6.18) 9066 (91.05)
Vehicle type (Vt)	1 = Motor vehicle 2 = Other	1542 (15.49) 8415 (84.51)
Driving status (Ds)	1 = Turning/overtaking/lane change 2 = Other	1154 (11.59) 8803 (88.41)
Vehicle safety status (Vss)	1 = Abnormal driving 2 = Normal	29 (0.29) 9928 (99.71)
Hazardous material (Hm)	1 = Dangerous goods 2 = Other	4 (0.04) 9953 (99.96)
Overloading (Ol)	1 = Overloading 2 = non-overloading	214 (2.15) 5688 (57.13)
Pavement alignment (Pa)	1 = Curved/Sloped segments 2 = Tangent segments	8717 (87.55) 1240 (12.45)
Separation (Sep)	1 = Nonseparation 2 = Separation	8243 (82.79) 1714 (17.21)
Traffic signal mode (Tsm)	1 = Uncontrolled 2 = Controlled	14 (0.14) 9943 (99.86)
Road safety rating (Rsr)	1 = Hazardous 2 = Normal	8348 (83.84) 1609 (16.16)
Road type (Rt)	1 = Highway 2 = Other	4779 (48.00) 5178 (52.00)
Roadside facility (Rf)	1 = non-protected 2 = Protective facilities	4855 (48.76) 5102 (51.24)
Road quality and condition (Rqc)	1 = Bad 2 = Normal	997 (10.01) 8761 (87.54)
Pavement structure (Ps)	1 = Poor 2 = Intact	1438 (14.44) 8519 (84.55)
Road segment type (Rst)	1 = Intersection 2 = non-intersection	2485 (24.96) 7472 (75.04)
Road hazard level (Rhl)	1 = Hazardous 2 = Normal	3408 (34.23) 6549 (65.77)
Road surface (Rs)	1 = Wet 2 = Dry	326 (3.27) 9631 (96.73)
Accident occurring period (Aop)	1 = Nighttime 2 = Daytime	1178 (11.83) 8779 (88.17)
Workday (Wd)	1 = Monday to Friday 2 = Weekend	7101 (71.32) 2856 (28.68)
Weather condition (Wc)	1 = Bad 2 = Good	2539 (25.50) 7418 (74.50)
Visibility (Vis)	1 = Low 2 = Good	1147 (11.52) 8810 (88.48)
Number of pedestrians (Ped)	0 1–3 3+	7984 (80.18) 1840 (18.48) 29 (0.29)
Number of vehicles (Mv)	0 1–3 3+	505 (5.07) 9221 (92.61) 96 (0.96)
Non-Motor vehicle (Nmv)	0 1–3	6483 (65.11) 3370 (33.85)
Type of accident (At)	1 = Vehicle-to-Vehicle 2 = Vehicle-Pedestrian 3 = Single-Vehicle	1278 (12.84) 8035 (80.70) 644 (6.47)

### Enhanced Multi-Task learning model

We introduced improved multi-task learning (MTL) deep neural network (Adv MT-DNN) methods that align weights to each task. First, we propose assigning task-specific weights based on their relative importance and difficulty. In traffic accident datasets, fatalities are typically less frequent but more critical, whereas property loss is more common. Therefore, we adjust the weights to ensure the model doesn’t ignore the minority tasks (fatalities) while still accounting for the more frequent ones (injuries and property loss). Specifically, higher weights can be assigned to the fatal task to ensure that the model gives more attention to learning from the less frequent but more impactful fatalities, while the injury and property loss tasks can receive lower but still significant weights to reflect their higher occurrence.

MTL involves leveraging the information contained in *m* learning tasks $\left\{ {{T_i}} \right\}_{{i=1}}^{m}$, where the tasks or a subset of them are related but not identical. Considering crash severities, there is a complex interdependency between fatalities, injuries, and property losses. There is a significant association among different severity tasks, and these multiple related tasks could assemble an MTL framework for better forecasting performance. By utilizing data from tasks, machine learning can improve a learner’s accuracy for each task^23^. In our proposed multi-task model framework, a shared layer is incorporated to learn common features across tasks, whereas the single-task learning Deep Neural Network (ST-DNN) trains each task independently, without shared parameters.

Given the strong interdependency between tasks, we introduce a shared loss for the common features learned across all tasks. This shared loss ${\ell _{shared}}$ is combined with the individual task losses, leading to a comprehensive model that learns shared and task-specific features. The shared learning layer can be represented as:

1 $${\ell _{total}}=\sum\nolimits_{{i=1}}^{m} {{w_i} \cdot {\ell _i}+\lambda } {\ell _{shared}}$$

where${\ell _i}$is the loss for the individual task *i*.${\ell _{shared}}$is the shared loss function that captures common features across tasks, encouraging the model to exploit task interdependencies. ${w_i}$is the weight for task *i*, and $\lambda$ is a hyperparameter controlling the trade-off between task-specific and shared learning.

The model also addresses the class imbalance within each task. For instance, within the fatal task, where fatalities represent a minority class, we incorporate a class-weighted loss function to increase the penalty for misclassifying fatalities. The class-weighted loss function for each task can be defined as:

2 $${\ell _{weighted}}= - \sum\nolimits_{{c=1}}^{C} {{w_c} \cdot {y_c}} +\log ({P_c})$$

*C* is the number of classes in the task.${w_c}$ is the class weight for class *c*, typically computed as the inverse of the class frequency:${w_c}=\frac{1}{{fr{e_{clas{s_c}}}}}$.${y_c}$ is the true label and ${P_c}$ is the predicted probability for class *c*.

Furthermore, the model utilizes task-specific loss functions that allow for individual tuning based on the difficulty and importance of each task. These loss functions are then combined with the task weights in the total loss function, allowing the model to adjust its learning focus during training. The individual task losses can be expressed as:

3 $${\ell _{total}}={\ell _{weighted,fatal}}+{\lambda _{fatal}} \cdot {\ell _{shared}}$$

4 $${\ell _{injury}}={\ell _{weighted,injury}}+{\lambda _{injury}} \cdot {\ell _{shared}}$$

5 $${\ell _{property}}={\ell _{weighted,property}}+{\lambda _{property}} \cdot {\ell _{shared}}$$

where$\ell _{{weighted,fatal}}^{{}}$, ${\ell _{weighted,injury}}$, and ${\ell _{weighted,property}}$ are the class-weighted loss functions for fatal, injury, and property loss tasks. ${\lambda _{fatal}}$, ${\lambda _{injury}}$, and ${\lambda _{property}}$ are hyperparameters that control the contribution of the shared loss to each task’s specific loss. Thus, the total loss function for an improved MTL model can be written as:

6 $${\ell _{total}}={\omega _{fatal}} \cdot {\ell _{fatal}}+{\omega _{injury}} \cdot {\ell _{injury}}+{\omega _{property}} \cdot {\ell _{property}}$$

${\ell _{fatal}}$, ${\ell _{injury}}$, and ${\ell _{property}}$ are the individual task loss functions for Fatal, Injury, and Property Loss, respectively. ${\omega _{fatal}}$, ${\omega _{injury}}$, and ${\omega _{property}}$ are the task-specific weights that reflect the relative importance and frequency of each task, notably, ${\omega _{fatal}}>{\omega _{injury}}>{\omega _{property}}$.

### Baselines

The Support Vector Machine with Fuzzy C-Means (SVM-FCM) combines Fuzzy C-Means (FCM) clustering with Support Vector Machines (SVM). First, FCM divides the data into distinct clusters based on features such as weather conditions, road type, and vehicle characteristics, with each data point having a degree of membership to multiple clusters. Then, an SVM model is trained for each cluster to classify the severity of the accidents^16^. Finally, the predictions from each SVM model are combined to yield a final classification, allowing the model to account for the complexities and nuances within different types of accident data. This approach enhances classification accuracy by leveraging fuzzy clustering to capture the subtle differences between accident categories and provides a more precise crash severity prediction.

Multilayer Perceptron (MLP) is a feedforward neural network consisting of multiple layers of neurons, where each neuron is connected to every neuron in the previous layer^24^. In traffic accident prediction, MLP models complex relationships between various factors contributing to accidents. It can effectively learn non-linear patterns in large and diverse datasets, making it suitable for predicting accident severity.

Light Gradient Boosting Machine (LightGBM) is an efficient framework for Gradient Boosting Decision Trees (GBDT)^25^. Its advanced techniques, such as histogram-based algorithms and leaf-wise tree growth, make it one of the fastest ensemble classifiers for large-scale traffic data. Its histogram-based algorithm achieves 60–80% memory reduction and 2.1× faster training speed compared to XGBoost’s pre-sorted methods^26^—critical for processing our 9,000 + sample dataset with 30 + heterogeneous features on NVIDIA V100 GPUs. The framework natively handles categorical variables through dedicated parameterization, eliminating one-hot encoding distortions while preserving crucial feature interactions. Empirical validation across transportation safety studies^27^ confirms LightGBM’s superiority in handling imbalanced crash data, demonstrating 12–18% higher F1 scores than CatBoost through leaf-wise growth strategies prioritizing high-error samples.

### Measure metrics

In keeping with the methodology utilized in other studies, we assessed our model using accuracy, precision, recall, and the Area Under Curve (AUC)^7^. AUC is the area under the ROC (Receiver Operating Characteristic) curve. The ROC curve plots the true positive rate (TPR) on the horizontal axis and the false positive rate (FPR) on the vertical axis. True Positive (TP) corresponds to positive categories correctly predicted as positive, while False Negative (FN) corresponds to positive categories incorrectly predicted as negative. Similarly, True Negative (TN) refers to negative categories correctly predicted as negative, and False Positive (FP) refers to negative categories incorrectly predicted as positive. The metrics are as follows:

7 $$ACC=\frac{{TP+TN}}{{TP+TN+FP+FN}}$$

8 $$TPR=\frac{{TP}}{P}=\frac{{TP}}{{TP+FN}}$$

9 $$FPR=\frac{{FP}}{N}=\frac{{FP}}{{FP+TN}}$$

10 $$precision=\frac{{TP}}{{TP+FP}}$$

11 $$recall=\frac{{TP}}{{TP+FN}}$$

### Model explanation

#### Selective Layer-Wise relevance propagation (SLRP)

Complex deep neural networks may be explained by Layer-wise Relevance Propagation (LRP)^28,29^. SLRP is a variant of the LRP method. Using localized propagation rules to backpropagate the output probability through the neural network systematically clarifies each input element’s contribution to that output probability^30,31^.


12


$R_{i}^{{(l)}}$ is the relevance score of the *i*^*th*^ node in layer *l*, $x_{i}^{{(l)}}$ is an input value of layer *l*, and $w_{{ij}}^{{(l,l+1)}}$ is the weight value between layer *l* and *l+*1. Here, $(g_{i}^{{(l)}}>0)$ denotes the sign of the gradient of the *i*^*th*^ activation in the *l* layer of the target class output. When the gradient is non-positive, the value of $(g_{i}^{{(l)}}>0)$ is set to 0, implying that the activation $x_{i}^{{(l)}}$ is included in the calculation. The relevant activation is incorporated and aids in the computing process if the gradient is positive. Lastly, + indicates that the values utilized are only positive^32,33^.

#### Shapley additive explanations (SHAP)

SHAP is a model-agnostic interpretation method that can elucidate the predictions of any ML model. It employs Shapley values to interpret box models, considers global and local levels, and aggregates Shapley values across all samples to determine the importance of features^21^.

13 $$impor\tan c{e_j}=\frac{1}{m}\sum\limits_{{t=1}}^{m} {\left| {\sigma _{j}^{{(i)}}} \right|} ,{\forall _j} \in FeatureSet$$

here, *m* represents the dataset’s sample size, while $\sigma$denotes the Shapley value of feature *j* in sample *i*.

#### Feature importance (FI-RF)

Random Forest (RF) is a popular machine learning technique composed of numerous individual decision trees, often utilized for ranking variable importance in traffic safety analysis^25^. Belonging to the bagging class of ensemble learning algorithms, RF combines multiple weak decision tree classifiers and makes final decisions through majority voting. The importance of a feature is calculated as the total decrease in impurity that the feature causes across all trees in the forest.

14 $$impor\tan c{e_j}=\frac{1}{T}\sum\limits_{{t=1}}^{T} {\sum\limits_{{i \in S_{j}^{t}}} {\frac{{{N_i}}}{m}\Delta i(i.t)} }$$

here, *T* represents the number of trees in the forest. $S_{j}^{t}$ denotes the set of all samples in the tree *t* where feature *j* is used for splitting. ${N_i}$ is the weight of the sample *i* and *m* is the total samples in the dataset. ${\Delta _i}(i,t)$ represents the decrease in impurity for sample *i* in tree *t* caused by feature *j*.

#### Local interpretable Model-agnostic explanations (LIME)

LIME is a model-agnostic method used to understand models’ decision-making processes^20^. It approximates the behavior of a black-box model by generating a local linear model around a specific data point, providing an interpretable explanation of the model’s output. LIME is useful for high-dimensional data and complex models, such as deep learning or ensemble methods.

15 $$impor\tan c{e_j}=\mathop {\arg \hbox{min} L(f,g,{\pi _j})}\limits_{{g \in G}} +\Omega (g)$$

where *j* is a sample, *g* is the local surrogate model, *G* is a set of simple local surrogate models, *f* is the black-box model, *L* is the loss function, ${\pi _j}$ is a proximity measure for the locality around *j*, $\Omega (g)$measures the local surrogate model complexity.

## Results and discussion

### Model performance

Regarding accuracy and AUC, on average, Adv MT-DNN outperformed ST-DNN by 6%, MLP by 8%, SVM-FCM by 7%, and LightGBM by 1% and outperformed SVM-FCM by an average of 7%, ST-DNN by 6%, MLP by 8%, and LightGBM by 1% (Tables 3 and 4). As for RTA-FS (Road Traffic Accident datasets, RTA), Adv MT-DNN demonstrates improvements across all types. SVM-FCM and ST-DNN demonstrate high stability across datasets with minimal impact from data distribution. At the same time, LightGBM and MLP are more sensitive to data imbalance, and Adv MT-DNN shows smaller variations, indicating strong robustness to balanced data. These results suggest that the model trained on the balanced dataset (BD-FS) has better discriminatory power and performs more effectively. To validate the effectiveness and superiority of MTL, the proposed model was compared with the single-task DNN model using the same training and test data. The Adv MT-DNN prediction model performs better than the ST-DNN in terms of ACC and AUC, highlighting the effectiveness of the multi-task method in capturing correlations between different severities. In addition, the metric fluctuation range of the Adv MT-DNN model is smaller than that of the single-task model, indicating that the multi-task model has better generalization ability.

**Table 3:** Mode performance comparison between balanced and RTA datasets.

Model	Metrics	BD-FS					RTA-FS				
		Injury	Fatal	No injury	Macro Avg	Macro AUC	Fatal	Serious	Slight	Macro Avg	Macro AUC
SVM-FCM^16^	ACC AUC	0.6332 0.7263	0.5811 0.7080	0.6373 0.7100	0.6172 0.7147	0.7291	0.4762 0.5331	0.5082 0.3051	0.5870 0.5864	0.5238 0.4749	0.4797
ST-DNN	ACC AUC	0.5861 0.7334	0.6112 0.7263	0.6843 0.7502	0.6272 0.7366		0.4652 0.5104	0.5842 0.4100	0.5993 0.4574	0.5495 0.4592	
MLP^24^	ACC AUC	0.6084 0.7312	0.5774 0.7163	0.6540 0.7491	0.6132 0.7322		0.4944 0.3080	0.5611 0.3874	0.5604 0.6344	0.5386 0.4433	
LightGBM^25^	ACC AUC	0.6443 0.6581	0.5743 0.6844	0.5760 0.6741	0.5982 0.6722		0.5010 0.4513	0.5242 0.4500	0.5403 0.6131	0.5218 0.5048	
Adv MT-DNN	ACC AUC	0.6661 0.7993	0.6494 0.7749	0.6904 0.7963	0.6686 0.7901		0.4963 0.4863	0.5881 0.4930	0.6500 0.5692	0.5781 0.5162	

**Table 4:** Enhanced model comparison between original, balanced, and public datasets.

Model	Dataset	Metrics	Fatal	Injury	No injury	Macro Avg	Final loss (Train)	Final loss (Val)
Adv MT-DNN	OD-FS	ACC AUC Pre Recall	0.4953 0.5963 0.5879 0.5877	0.5880 0.6790 0.6650 0.6588	0.6507 0.7047 0.7013 0.7242	0.5780 0.6600 0.6514 0.6569	0.6892	0.6534
Adv MT-DNN	BD-FS	ACC AUC Pre Recall	0.6494 0.7749 0.7047 0.7116	0.6661 0.7993 0.7409 0.7476	0.6904 0.7963 0.7732 0.7698	0.6686 0.7901 0.7396 0.7430	0.6513	0.6121
Adv MT-DNN	RTA-FS	ACC AUC Pre Recall	0.4963 0.4863 0.5101 0.4527	0.5881 0.4930 0.5734 0.4382	0.6500 0.5692 0.5743 0.3485	0.5781 0.5162 0.5526 0.4131	0.6454	0.6378

**Fig. 2:**
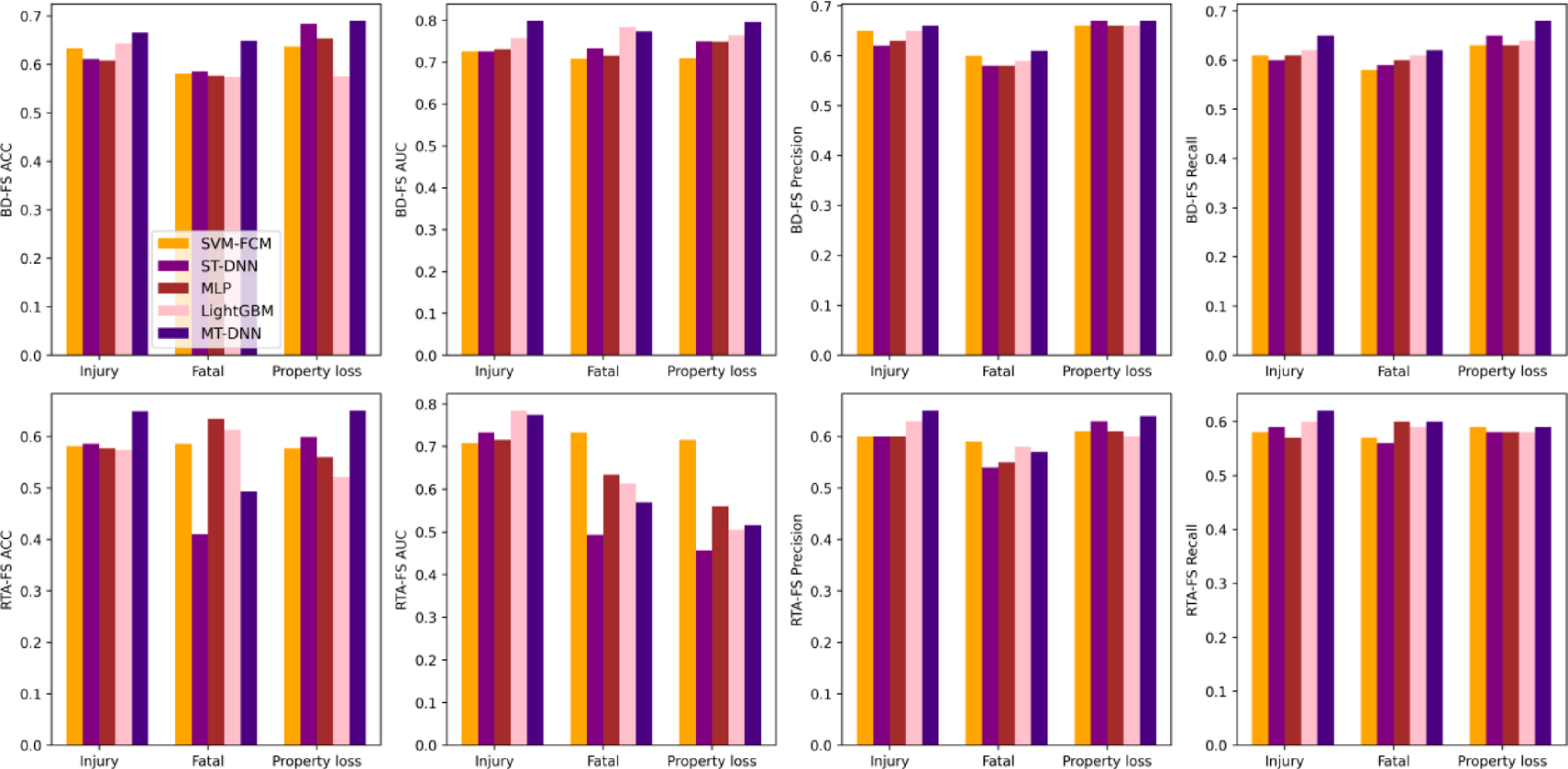
Model results between balanced and RTA datasets.

Additionally, Adv MT-DNN shows a consistent performance across the metrics, with higher scores on the BD-FS and the OD-FS (Original Dataset) (Fig. 2). The model performs best on the BD-FS dataset across all metrics, particularly in AUC and Precision, where the score reaches about 0.65–0.69. ST-DNN, on the other hand, generally performs well but lags behind Adv MT-DNN in all metrics, especially in ACC and AUC on the BD-FS dataset. It shows some lower values, particularly in Recall and Precision. In the category-specific results, both models perform similarly, with Adv MT-DNN outperforming ST-DNN across all severity categories, especially in terms of Property Loss and Fatal. This highlights the advantage of multi-task learning in improving the performance of minority classes.

Table 5 shows the hyperparameter settings. During the training phase, we incorporated two techniques to mitigate the risk of overfitting on the training dataset: (i) Validation Set. After each epoch, we randomly selected 20% of the training dataset as a validation set. This set was employed to monitor the model’s performance during training and to prevent overfitting by ensuring generalization to unseen data. (ii) Early Stopping with Patience. We applied the early stopping with patience technique, which involves monitoring the model’s performance on the validation set. If there was no improvement or a decline in the model’s performance on the validation set for 10 consecutive epochs, the training was halted early.

**Fig. 3:**
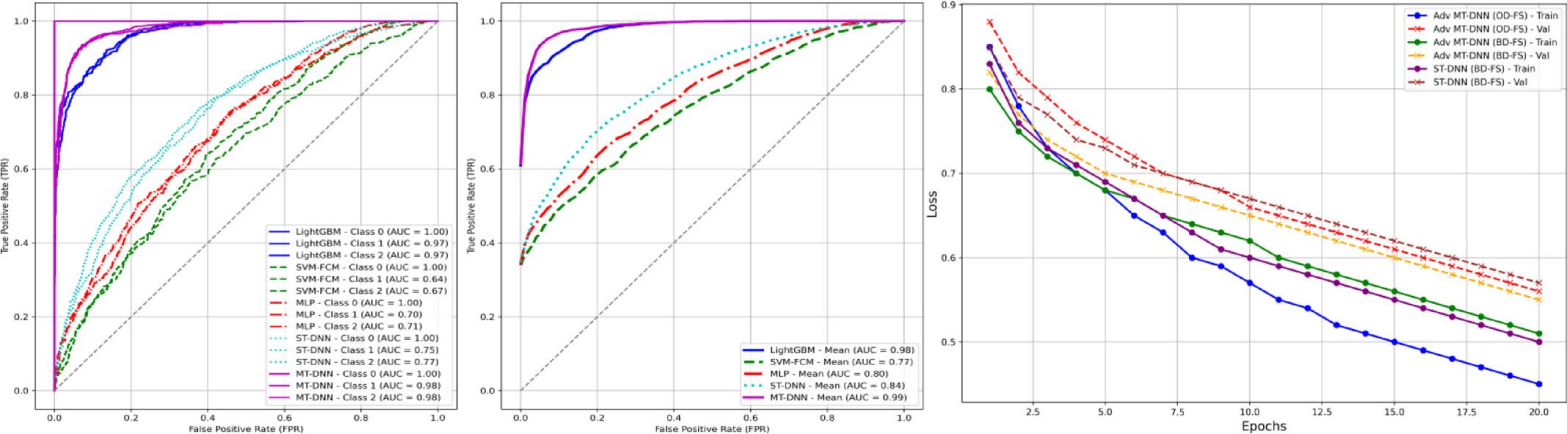
**(a)** ROC curves per class and mean and **(b)** epochs process.

Two types of evaluations were conducted: per-class ROC curves and mean ROC curves across all classes. Figure 3(a) displays the ROC curves for each class (0, 1, and 2) and illustrates how well each model distinguishes between classes (i.e., no injury, severe, and fatal injury) at different classification thresholds. Figure 3(a) presents the average ROC curves for each model calculated by combining the individual ROC curves of all classes. It demonstrates consistently superior performance in distinguishing between serious and minor injuries. In other models, ROC curves exhibit slight overlaps at certain points, suggesting a slightly less effective ability to discriminate between these classes. The Adv MT-DNN (BD-FS) model demonstrates strong performance on the training and validation sets, with relatively low and consistent loss values, suggesting it may be the most effective model among the three (Fig. 3(b)). In contrast, the Adv MT-DNN (OD-FS) model exhibits higher validation loss than training loss, indicating a potential risk of overfitting. Lastly, the ST-DNN (BD-FS) model shows relatively higher loss values, implying that its performance on the training and validation sets is inferior to the other two models.

**Table 5:** Model hyperparameter settings.

Model	Hyperparameters	Value
SVM-FCM	c m	4 1.5
ST-DNN	hidden_layers learning_rate batch_size	2 0.02 32
MLP	hidden_layers learning_rate dropout	2 0.02 0.2
LightGBM	boosting_trees learning_rate max_depth	100 0.01 6
MT-DNN	hidden_layers learning_rate dropout batch_size	4 0.02 0.2 32

### Case studies

The case studies from the BD-FS compare models based on the top five factors influencing the results, as shown in Tables 6 and 7. In the ‘Injury’ prediction, all models made correct classifications. However, for the ‘Fatal,’ only the MT-DNN and MLP provided accurate predictions, while the other baselines failed. In the ‘Property Damage’ category, MT-DNN was the sole model to offer correct predictions within the range of 1,000 to 9,999 yuan, whereas other models either underperformed or failed to deliver precise results. This finding reveals that the latter two models have higher features with missing values. Additionally, the explanations provided by SVM-FCM and MLP lack the in-depth detail offered by MT-DNN and ST-DNN.

**Table 6:** Case 1—top 5 features generated by prediction models.

Model	MT-DNN	ST-DNN	MLP	LightGBM	SVM-FCM
Property losses (yuan)	1,000–9,999	1,000–9,999	1,000–9,999	1,000–9,999	1,000–9,999
	☑	☒	☒	☒	☒
Top1	Bac (no)	Sdu (seatbelt non-use)	Gd (unknown)	Rsr (unknown)	Pa (unknown)
Top2	Rqc (bad)	Sep (unknown)	Rf (nonprotected)	Rs (wet)	Rqc (bad)
Top3	Aop (nighttime)	Pa (straight)	Ps (poor)	De (under 10 years)	Rf (nonprotected)
Top4	Rs (wet)	Aop (nighttime)	Vis (low)	Pa (straight)	Aop (nighttime)
Top5	De (under 10 experience years)	Rs (wet)	De (unknown)	Bac (no)	Vis (low)
Number of injuries	10–19	1	2	1	1
	☑	☑	☑	☑	☑
Top1	Bac (no)	Rst (intersection)	Bac (no)	De (under 10 experience years)	Gd (unknown)
Top2	Aop (nighttime)	Ag (unknown)	Rt (highway)	Ag (unknown)	De (unknown)
Top3	Rqc (bad)	Sdu (seatbelt non-use)	Rf (nonprotected)	Gd (unknown)	Aop (nighttime)
Top4	Pa (straight)	Wd (no)	Rst (intersection)	Rqc (bad)	Vt (motor)
Top5	Rs (wet)	Gd (unknown)	Rsr (hazardous)	Vss (poor braking)	Tsm (unknown)
Number of fatalities	2	0	2	0	1
	☑	☒	☒	☒	☒
Top1	Rs (wet)	Ag (unknown)	Aop (nighttime)	Bac (no)	Gd (unknown)
Top2	Aop (nighttime)	Rt (highway)	Rst (intersection)	Sdu (seatbelt non-use)	Rqc (bad)
Top3	Rqc (bad)	Rf (nonprotected)	Rf (nonprotected)	Aop (nighttime)	De (unknown)
Top4	Pa (straight)	Rst (intersection)	Vis (low)	Ol (no)	Bac (no)
Top5	Bac (no)	Rs (wet)	Bac (no)	Rf (no)	Aop (nighttime)

**Table 7:** Case 2—top 5 features generated by prediction models.

Model	MT-DNN	ST-DNN	MLP	LightGBM	SVM-FCM
Property losses (yuan)	1,000–9,999	1,000–9,999	1,000–9,999	1,000–9,999	1,000–9,999
	☑	☒	☒	☒	☒
Top1	Bac (no)	De (unknown)	Rf (nonprotected)	Rsr (hazardous)	Gd (male)
Top2	Rf (nonprotected)	Ag (unknown)	Bac (no)	Ag (unknown)	Rt (highway)
Top3	Pa (straight)	Vis (good)	Rs (dry)	Rt (highway)	Rf (nonprotected)
Top4	Rt (highway)	Rf (nonprotected)	Rsr (hazardous)	Rf (nonprotected)	De (under 10 experience years)
Top5	De (under 10 experience years)	Rt (highway)	Rst (intersection)	Aop (daytime)	Vt (motor)
Number of injuries	1	1	1	1	1
	☑	☑	☑	☑	☑
Top1	Rst (intersection)	Vt (motor)	Rqc (unknown)	De (under 10 years)	Gd (male)
Top2	Rf (unknown)	Sdu (unknown)	Bac (no)	Tsm (unknown)	De (unknown)
Top3	Rt (highway)	Ag (unknown)	Wc (good)	Gd (male)	Aop (daytime)
Top4	Ol (no)	Wd (yes)	Sdu (unknown)	Aop (unknown)	Wd (yes)
Top5	Rqc (bad)	Aop (daytime)	Rf (nonprotected)	Wd (no)	Sdu (unknown)
Number of fatalities	1	0	0	0	0
	☑	☒	☒	☒	☒
Top1	Rf (nonprotected)	Ag (unknown)	Bac (no)	Wd (no)	Wd (no)
Top2	Pa (straight)	Rsr (hazardous)	Pa (straight)	Aop (daytime)	De (unknown)
Top3	Bac (no)	Rf (unknown)	Rqc (intact)	De (unknown)	Aop (daytime)
Top4	Rqc (intact)	Rst (intersection)	Rt (highway)	Rf (nonprotected)	Rsr (hazardous)
Top5	Rst (intersection)	Rs (dry)	Ol (no)	Rsr (unknown)	Gd (male)

### Insights from explanation methods

#### Explainability comparison

Table 8 lists the top five features of explanation methods. Vss (Vehicle safety status) frequently appears in the SHAP and FI-RF results for ‘Property Loss,’ while Sdu ranks highly in SHAP for ‘Property Loss’ and appears in FI-RF for ‘injury.’ Road conditions, such as Rqc (bad roads), Rs (wet roads), and Rsr (normal), are recurring features across all methods. Each explainer has its unique focus and strengths. FI-RF prioritizes traffic-related and infrastructural features, such as Rst (Road section type) and Vt (Vehicle type) for ‘Fatal,’ and Rst and Tsm (Traffic signal mode) for ‘Property Loss,’ reflecting its focus on physical and structural conditions. LIME provides local, interpretable explanations for specific instances, focusing on individual behaviors and contextual factors. For example, LIME identifies the driver’s age as a factor influencing ‘Injury’ accidents, showing its sensitivity to driver demographics. It also highlights key features such as driver experience, traffic lights, road markings, and vehicle safety status as important factors across all severity categories. While LIME provides local insights into the contributions of individual features, its interpretations are typically more instance-specific rather than offering a comprehensive, global understanding. SLRP places heavy emphasis on environmental and road conditions, highlighting factors such as Rqc (bad roads), which ranks at the top for both ‘Fatal’ and ‘Injury,’ and Pa (straight roads), which is significant for ‘Injury’ and ‘Property Loss.’ It also emphasizes that the risks of nighttime driving are consistently identified across all severity categories, indicating the importance of environmental context in accident severity. Therefore, SHAP is the most effective explainer and it identifies unique key predictors for interpretable insights into specific feature values and their contributions to predictions.

**Table 8:** Explanation methods comparison.

Severity	Ranking	SLRP	SHAP	FI-RF	LIME
Fatal	Top1	Rqc (bad)	Bac = 2 (high alcohol content)	De	Ag$\:\le\:$29 (age under 29)
	Top2	Aop (nighttime)	Vss = 1 (abnormal driving)	Rst	5$\:<$Tsm$\:\le\:$45 (road markings, traffic lights)
	Top3	Rs (wet)	Sdu = 6 (absence of safety device usage)	Vt	De$\:\le\:$8 (driver under 8 experience years)
	Top4	Pa (straight)	Aop = 3 (nighttime)	Pa	Da$\:\le\:$0 (nonuse)
	Top5	Bac (nonuse)	Rhl = 2 (normal)	Sep	Wc$\:\le\:$1 (bad)
Injury	Top1	Bac (nonuse)	At = 2 (vehicle-pedestrian)	Rt	Ag$\:\le\:$29 (age under 29)
	Top2	Aop (nighttime)	Rsr = 2 (normal)	Sdu	Ed$\:>$70 (other)
	Top3	Rqc (bad)	Aop = 3 (nighttime)	De	5$\:<$Tsm$\:\le\:$45 (road markings, traffic lights)
	Top4	Rs (wet)	Mv = 2 (two motor vehicles)	Ag	Rst$\:\le\:$21 (intersection)
	Top5	Pa (straight)	De = 13 (driver with 13 experience years)	Rsr	De$\:\le\:$8 (driver under 8 experience years)
Property loss	Top1	Bac (nonuse)	Sdu = 6 (absence of safety device usage)	Vss	Wc$\:\le\:$1 (bad)
	Top2	Sep (nonseparation)	Vss = 1 (abnormal driving)	De	Ag$\:\le\:$29 (age under 29)
	Top3	Rf (nonprotected)	Aop = 3 (nighttime)	Sep	5$\:<$Tsm$\:\le\:$45 (road markings, traffic lights)
	Top4	Pa (straight)	De = 13 (driver with experience 13 years)	Tsm	Rst$\:\le\:$21 (intersection)
	Top5	Aop (nighttime)	Ed = 60 (secondary or vocational education)	Wd	Da$\:\le\:$0 (nonuse)

#### Local explanation

**Fig. 4:**
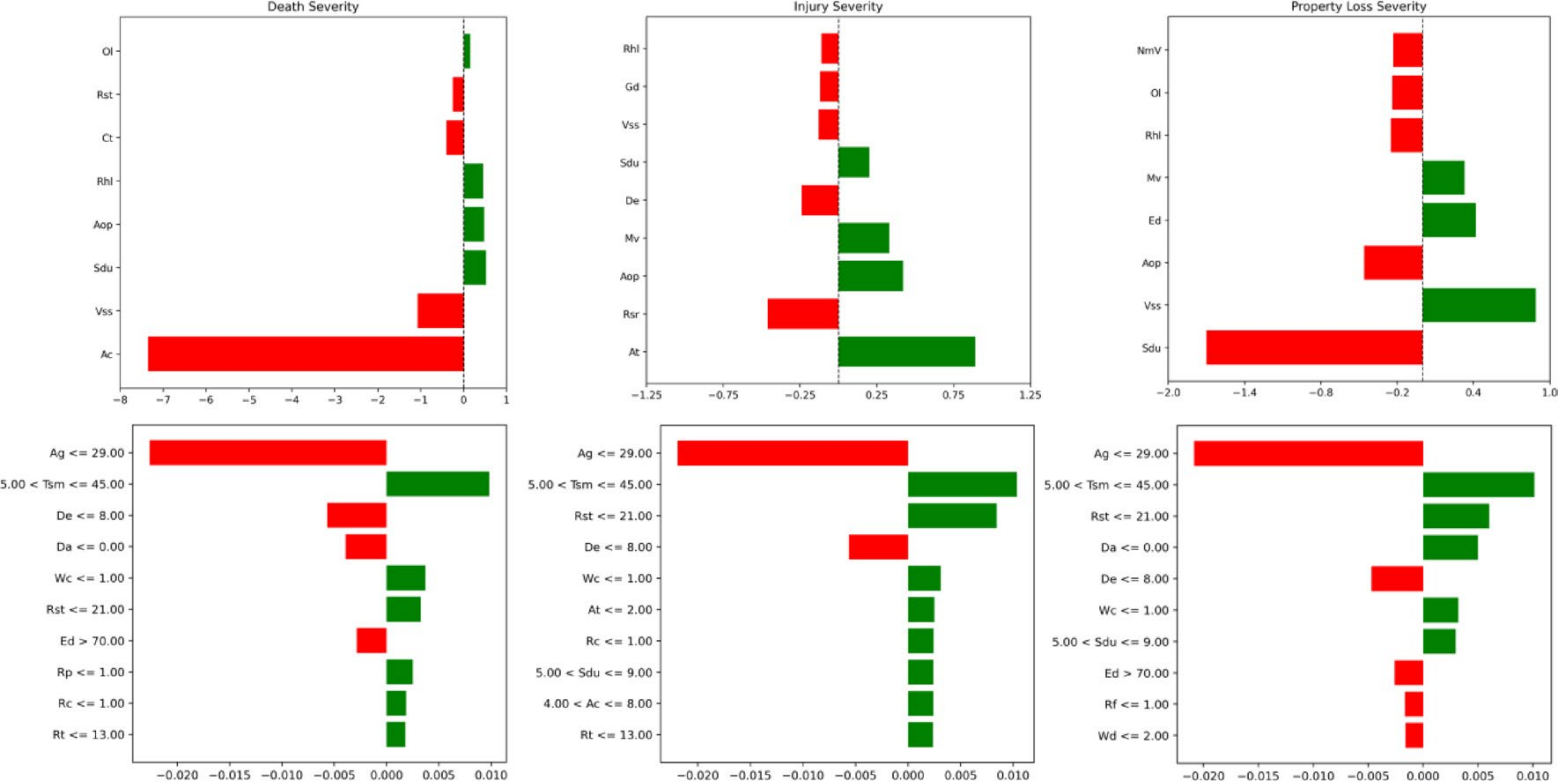
Local explanation comparison with SHAP and LIME.

Figure 4 shows the local explanation of the SHAP and LIME explainer across three types: death, injury, and property loss severity. The x-axis represents the contribution of each feature, while the y-axis corresponds to the feature values for individual instances. SHAP provides consistent global explanations with a direct contribution value for each feature, while LIME presents local explanations using bar lengths and colors to indicate the feature’s contribution and impact direction. The SHAP explainer provides consistent interpretations for individual samples, specifically, with five key features identified for death severity and six features for injury accidents. Regarding property loss, the most significant factors identified are Sdu (Safety device usage), Vss (Vehicle safety status), and De (Driver experience). In contrast, the LIME explainer represents each feature as a bar, with the length of the bar indicating the contribution of that feature to the prediction of death severity. The color of the bars often reflects the direction of impact, with some features contributing positively to the likelihood of death (increasing severity) and others having a mitigating effect (decreasing severity). Specifically, the most influential features are related to factors such as age ($\:\le\:$29), weather conditions ($\:\le\:$1), traffic infrastructure, and environmental conditions (5$\:<$Tsm$\:\le\:$45, Rst$\:\le\:$21), and the driver’s characteristics, such as having less than 8 years of driving experience, no drug use (De$\:\le\:$8, Ed$\:>$70, Da$\:\le\:$0).

#### Feature contribution

**Fig. 5:**
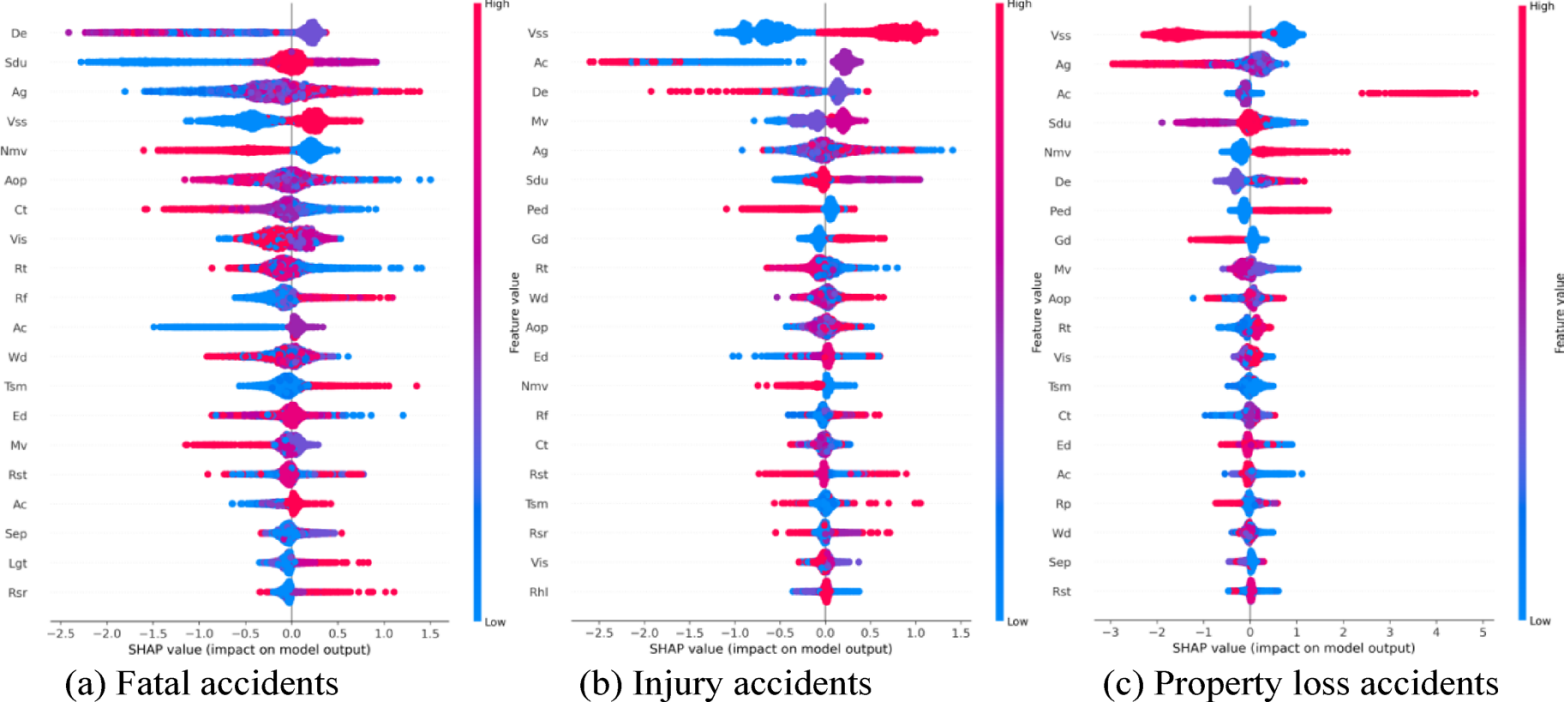
SHAP values.

Figure 5 illustrates the SHAP values corresponding to the three levels of crash severity. The x-axis represents the SHAP values, where values greater than 0 indicate a positive contribution to the severity of the crash, while values less than 0 reflect a negative contribution. The color gradient, ranging from blue to red, represents low to high feature values. For slight injuries, the focus is on SHAP values to the left of the 0-axis, indicating features that reduce the likelihood of more severe outcomes. Conversely, for fatal injuries, the key results lie to the right of the 0-axis, highlighting features that increase severity. Notably, characteristics such as age (Ag), road facilities (Rf), traffic signal mode (Tsm), blood alcohol content (Bac), and pavement alignment (Pa) have a stronger influence on fatal severity—higher values of these features are associated with more severe outcomes. Additionally, alcohol content (Bac) and driver experience (De) have a significant impact on injury severity, while factors like vehicle safety status (Vss), age (Ag), and alcohol content (Bac) strongly affect the severity of property loss.

(1) Road attributes.

The road type (Rt) is one of the most important factors influencing accident outcomes^34^. Other features, such as weather conditions (Wc), road facility (Rf), and visibility (Vis), directly affect the likelihood of injury and death severity^35^. For instance, poor visibility conditions (Vis = 1) increase the probability of death, while roadside facilities (Rf = 2) reduce the likelihood of injury severity. Motor vehicles (Mv) and road hazard levels (Rhl) also play a role, as their impact varies across different accident outcomes.

(2) Driver attributes.

**Fig. 6:**
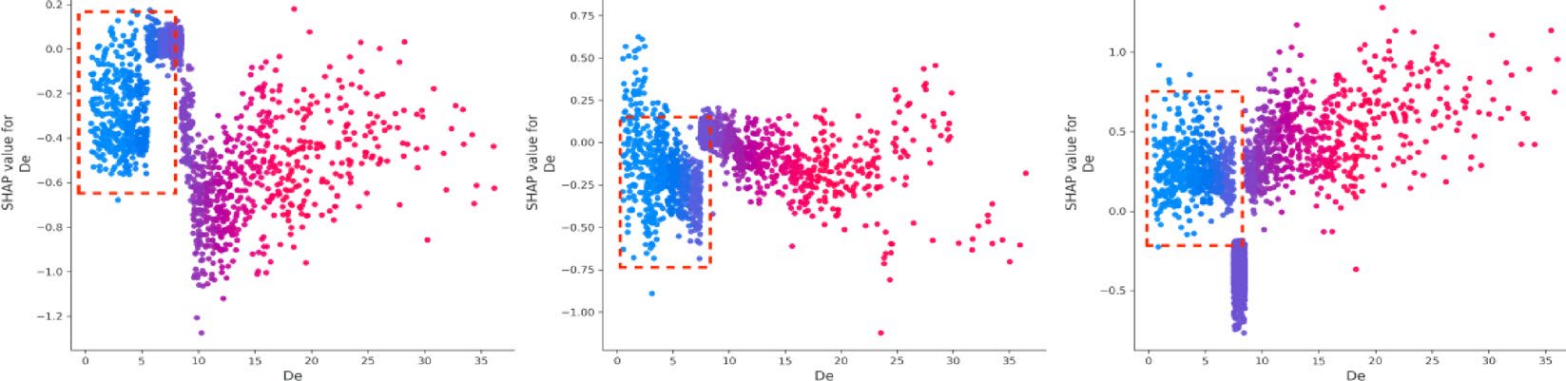
SHAP values for driver experience (De).

**Fig. 7:**
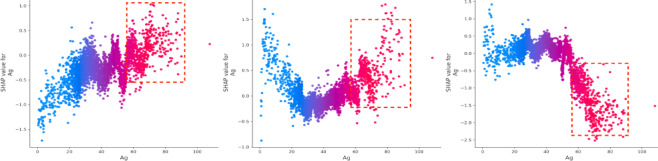
SHAP values for age (Ag).

**Fig. 8:**
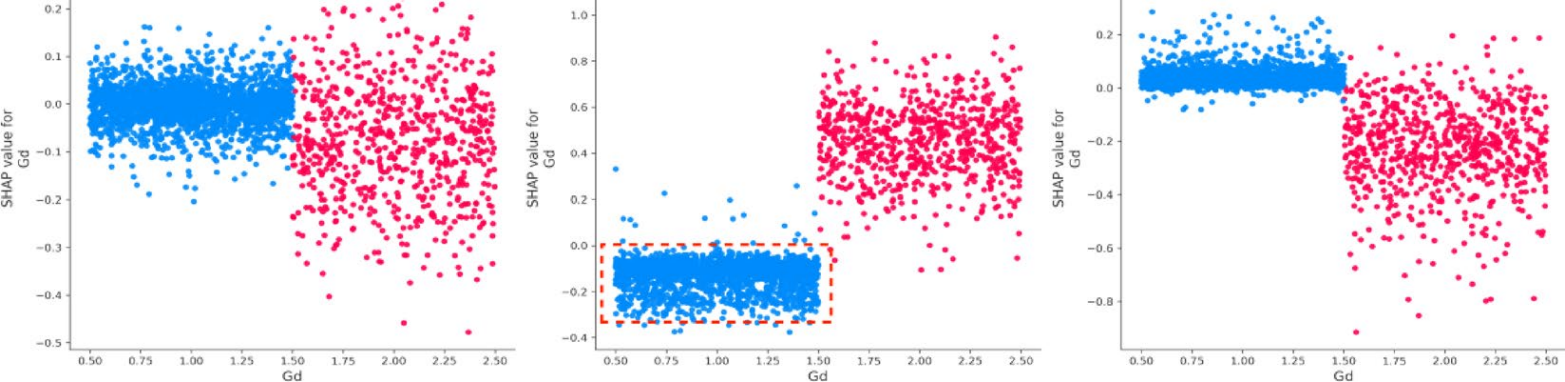
SHAP values for gender (Gd).

**Fig. 9:**
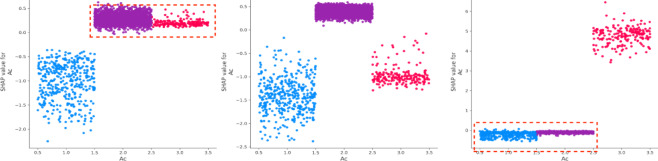
SHAP values for drunk driving (Bac).

The vehicle safety status (Vss) is a critical factor, consistently ranking among the top two contributors to accident outcomes. Driver experience (De) has a substantial negative impact on death severity. On the other hand, driver age (Ag) significantly affects property loss, with older drivers contributing more to this outcome^36,37^. Drivers with less experience tend to perform worse in both death and injury severity cases, though they have a more favorable impact in cases involving property loss. Additionally, while older drivers are more commonly involved in death and injury accidents, they tend to have the opposite effect on property loss (Figs. 6 and 7). Gender (Gd) appears to be more stable in its effect compared to driver experience (De) and age (Ag) (Fig. 8). Male drivers (Gd = 1), however, have a negative influence on the model’s outcome^38^. The type of accident (Bac) also plays a key role in increasing injury severity, highlighting the importance of driver-related characteristics^39^. Low alcohol levels (Bac = 1) are associated with a reduced impact on the model, while higher alcohol content (Bac = 2,3) is positively correlated with death severity (Fig. 9)^40,41^.

(3) Vehicle characteristics.

**Fig. 10:**
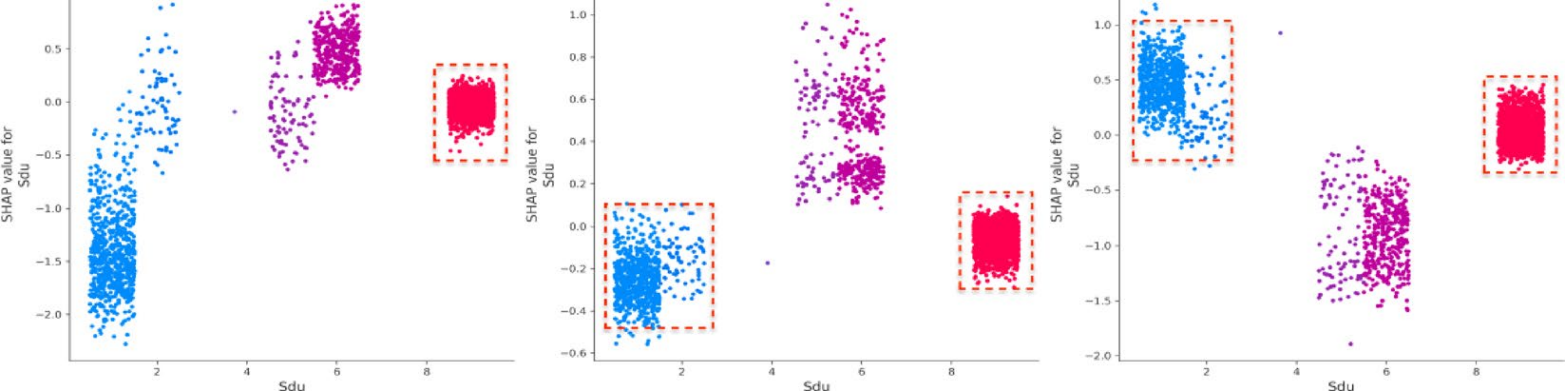
SHAP values for safety device usage (Sdu).

The condition of the road surface (Sdu) has a strong influence, significantly increasing both death and injury severity while reducing the likelihood of property loss^41,42^ (Fig. 10). The presence of traffic control mode (Tsm) positively impacts both injury severity and property loss. Vehicle safety status (Vss) plays a dominant role in determining property loss, emphasizing the critical influence of vehicle-related factors^43^.

(4) Environment and temporal attributes.

**Fig. 11:**
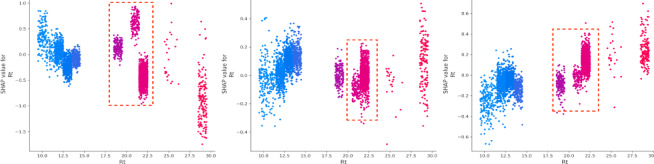
SHAP values for road type (Rt).

**Fig. 12:**
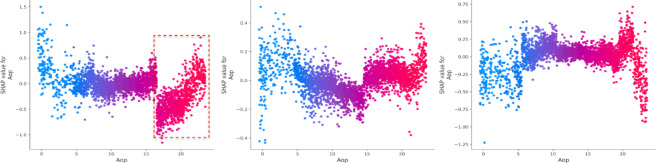
SHAP values for accident occurring period (Aop).

Lastly, factors such as the number of non-motor vehicles (Nmv), time of the day (Aop), and pedestrian involvement (Ped) exhibit distinct effects on accident outcomes^44^. The number of vehicles (Mv) has a significant effect on increasing property loss, while pedestrian involvement varies in its influence across different severity levels^45^. Road type is also affecting crash severity (Fig. 11). The SHAP values within the red box are relatively stable and high when the road type is an urban expressway or a general urban road (Rt = 21,22). This suggests that these road types consistently contribute positively to the prediction of death severity, indicating a higher risk of fatal accidents. For injury severity, the SHAP values are also positive, though slightly lower compared to the plot on the left. According to Fig. 12, we can conclude that Aop shows significant non-linear patterns in SHAP values across all severity categories. Early (0–6) and late hours (18–24) are high-impact periods where Aop contributes most to accident severity, especially for fatal and injury categories.

#### Interaction effects

The x-axis displays the SHAP interaction values and represents the interaction effect of two factors on the current level. A SHAP interaction value greater than 0 indicates a positive effect, while values less than 0 indicate negative effects. For injury severity and property loss accidents, results to the left of the 0-axis are of interest, while for fatal accidents, attention should be on the right of the 0-axis. Several meaningful interaction effects associated with accidents have been identified and discussed below.

(1) Interaction effects among important environmental attributes.

**Fig. 13:**
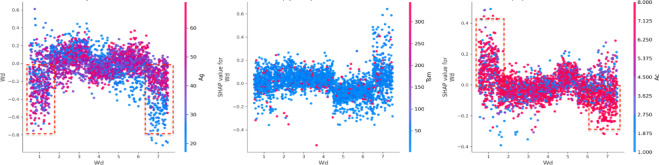
Interaction effects between workday (Wd) and road type (Rt).

**Fig. 14:**
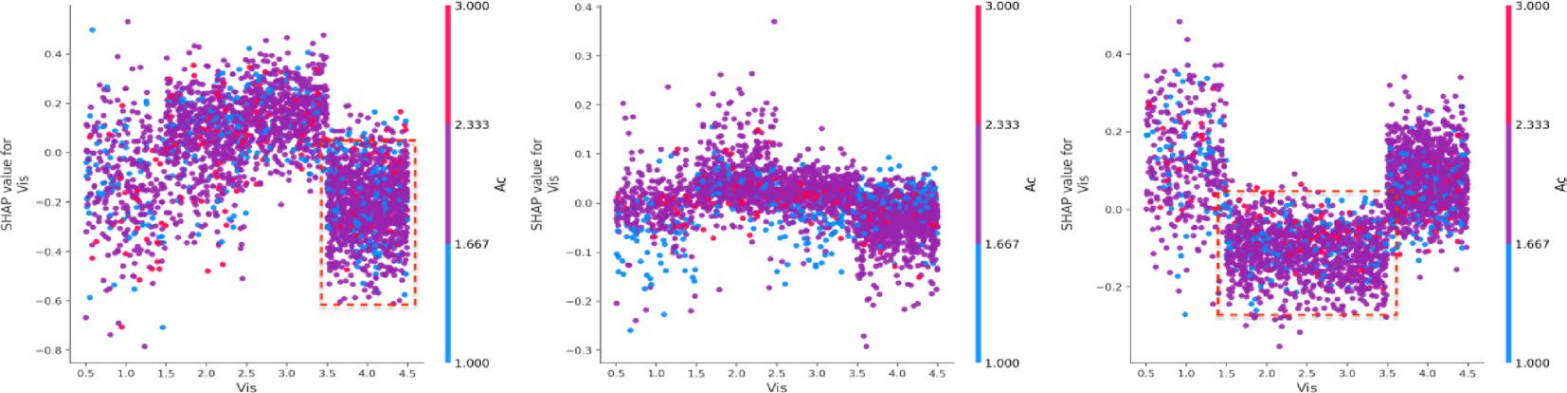
Interaction effects with visibility (Vis) and drunk driving (Bac).

For death severity, when the workday (Wd) value approaches a value of 6 (Saturday), there is a clear downward trend in the SHAP value^46,47^. This suggests that during heavy snow, Wd negatively influences property loss^48^. However, as Wd increases to 7 (Sunday), the SHAP value shifts positively (Fig. 13). When it is a weekend (Wd = 6,7), driving under the influence (Bac = 1,2,3), the property loss is negative, while it is Monday (Wd = 1), and under the same Bac condition, the SHAP values shift positively. For fatal severity, whether it is workday and age under 45 (Ag < 45), the SHAP values are consistently negative. Figure 14 shows a distinct cluster of points where the SHAP values are mostly positive for lower visibility conditions (Vis = 2). When visibility is less than 100 m (Vis < 3), the SHAP values are largely negative, indicating a decreased risk of property loss. However, as visibility increases to 200 m or more (Vis > 3), the SHAP values shift to positive, with this positive contribution becoming more pronounced at higher visibility^49,50^.

(2) Interaction effects among important driver attributes.

**Fig. 15:**
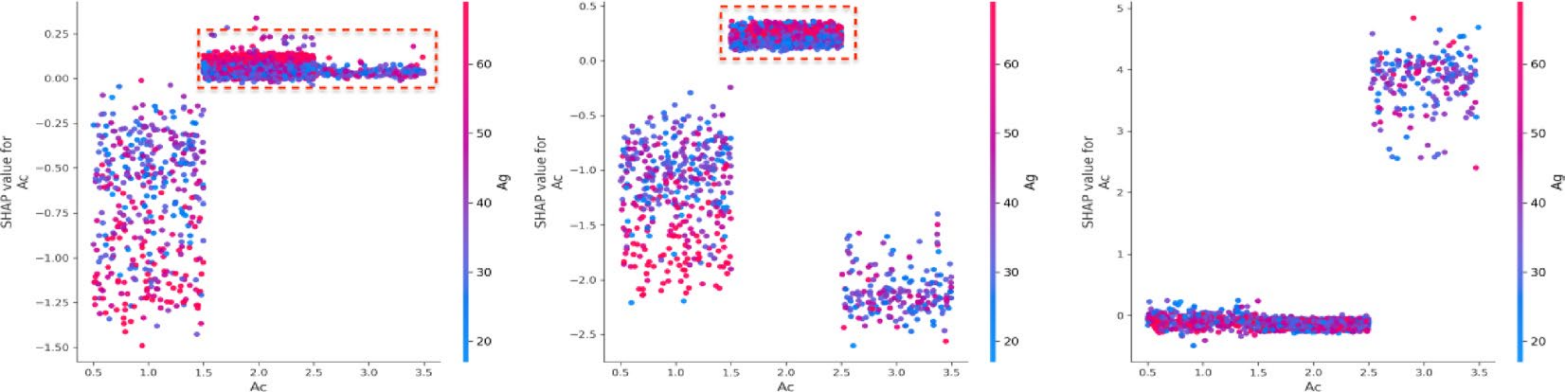
Interaction effects with alcohol content (Bac) and age (Ag).

**Fig. 16:**
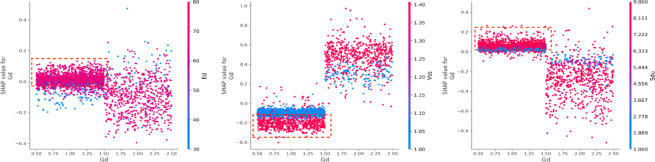
Interaction effects between gender (Gd) and driver attributes.

Across all three accident outcomes (death, injury, and property loss), the variable ‘Bac (Alcohol content) exhibits a non-linear relationship with predicted values^40^. Lower values of Bac generally contribute negatively, while higher values have a positive influence (Fig. 15). Specifically, for death and injury severity, SHAP values are negative when the driver is involved in drunk driving (Bac = 1). However, when the alcohol content exceeds 79 (Bac = 2), SHAP values become positive. While drivers age over 50, and driving under the influence (Bac = 3), the SHAP values are positive. Gender (Gd) is another significant driver characteristic (Fig. 16)^36^. When the driver is male (Gd = 1) and has a secondary or vocational education (50 < Ed < 60), the interaction effect is positive, indicating that both factors are positive to the model’s output. Conversely, when poor braking (Vss = 1) interacts with male drivers (Gd = 1), the SHAP values are negative, showing an opposing effect. Additionally, when male drivers (Gd = 1) do not use safety devices (Sdu = 6), there is a positive impact on property loss^43^.

(3) Interaction effects among important vehicle attributes.

**Fig. 17:**
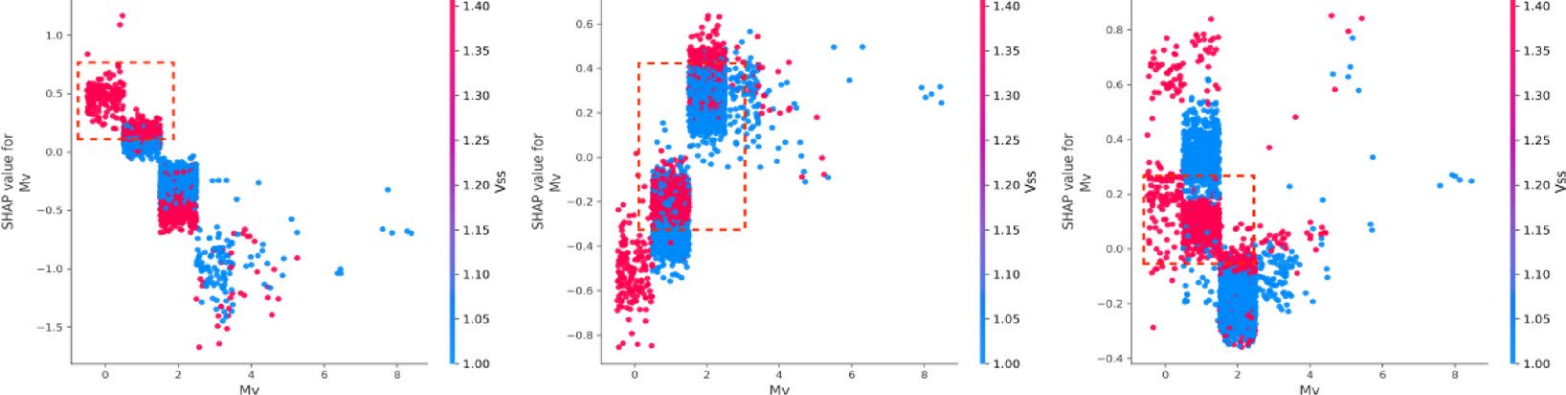
Interaction effects between motor vehicle (Mv) and vehicle safety status (Vss).

**Fig. 18:**
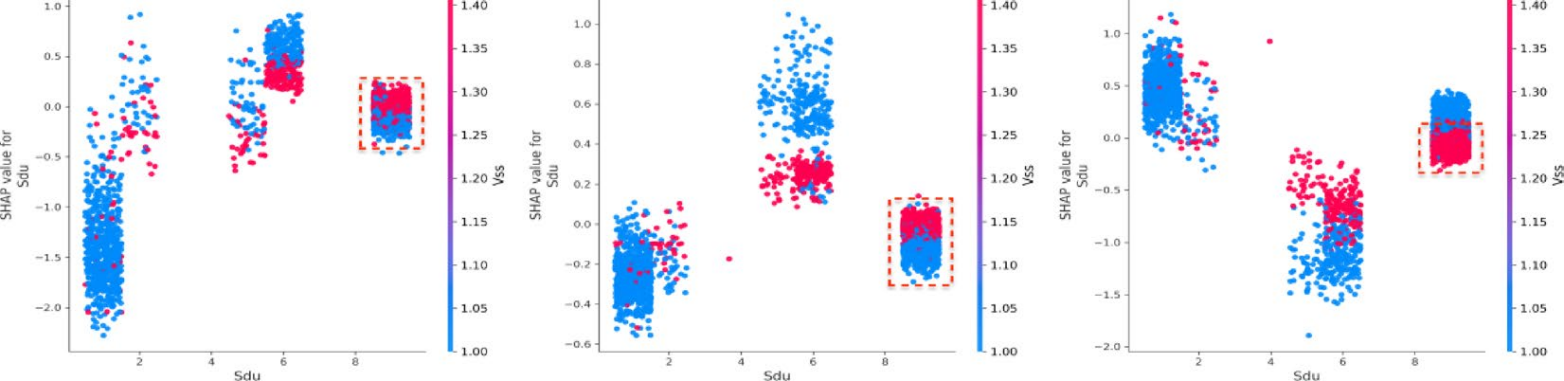
Interaction effects between safety device (Sdu) and vehicle safety status (Vss).

As one of the top three important factors, vehicle safety status (Vss) plays a crucial role in vehicle-related outcomes^35^. The number of vehicles involved strongly correlates with vehicle safety status. When two vehicles are involved (Mv = 2), vehicle safety status significantly impacts all three types of accidents. Notably, in non-motor vehicle accidents (Mv = 0), vehicles are under safety conditions and have a positive effect (Fig. 17). In cases where only one vehicle is involved (Mv = 1), such as in a collision with pedestrians or cyclists, Vss (Vss = 1) positively influences death and property loss, while the absence of safety vehicles negatively affects injury severity. The interaction between safety device usage (Sdu) and vehicle safety status (Vss) reveals a negative impact on death severity, especially when safety devices are non-use (Sdu = 9) and there are poor braking conditions (Vss = 1). Additionally, the relationship between Sdu and Bac shows that combining seatbelt usage (Sdu = 1) with driving under the influence results in a positive prediction for property loss. Conversely, when the alcohol content is low (Bac < 2) and seatbelts are used (Sdu = 1), the prediction for injury severity exhibits a counteracting effect (Fig. 18).

### Risk factors for accident and intervention strategies

Table 9 summarizes the key feature contributions and interaction effects. Critical factors such as workday, road type, visibility, blood alcohol content, and safety device usage are highlighted. The combination of visibility and blood alcohol content indicates that, even under high visibility conditions, drivers with zero undetectable Bac may still face increased fatality risks. This could be attributed to overconfidence or risky driving behaviors, where the absence of alcohol may lead drivers to feel overly secure, encouraging unsafe decisions on the road. The absence of safety devices (e.g., seat belts and helmets) is another critical factor that significantly exacerbates the risks of severe accidents. Male drivers and older drivers, especially in adverse conditions such as poor visibility or when driving under the influence of alcohol, are disproportionately involved in severe accidents. Additionally, the interaction between lower education levels (e.g., vocational education) and male drivers suggests a potential lack of safety awareness, further increasing the risk of fatal accidents. Drivers with lower education levels might not fully appreciate the dangers associated with poor driving behaviors or might be less likely to adopt safety precautions. Lastly, weekend and urban road types contribute to a higher fatality rate. These conditions, which often reduce road traction, impair visibility, and create more complex driving environments, significantly elevate the risk of severe accidents, particularly when compounded by factors such as alcohol consumption or lack of safety devices. These findings emphasize the need for several key strategies to reduce accident severity: enforcing stricter alcohol limits, promoting the use of safety devices, and enhancing driver training (especially for younger and older drivers) to mitigate risks associated with adverse weather conditions.

**Table 9:** Feature contribution and interaction affect each severity.

	Fatal	Injury	Property loss
Feature contribution	Weekend (Wd)$\:=$6,7		
	Urban expressway roads and general urban roads (Rt)$\:=$21,22		—
	—	Visibility of 100–200 m (Vis)$\:=$2	Visibility below 100 m (Vis)$\:<$3, visibility of 200 m and more (Vis)$\:>$3
	Driving under the influence of alcohol (Bac)$\:=$1,2,3		—
	Drivers aged between 60 and 80 (60$\:<$Ag$\:<$80)		
	Drivers with 10 years or less of experience (0$\:<$De$\:<$10)		
	Male drivers (Gd$\:=$1)		
	Early (0$\:<$Aop$\:<$6) and late hours (18$\:<$Aop$\:<$24)		
Interaction effects	Weekend (Wd)$\:=$6,7 & driving under the influence of alcohol (Bac)$\:=$1,2,3	Weekend (Wd)$\:=$6,7 & driver age under 45 (Ag)$\:<$45	Monday (Wd)$\:=$1 & driving under the influence of alcohol (Bac)$\:=$1,2,3
	Male drivers (Gd)$\:=$1 & Secondary or Vocational education (Ed)$\:=$3	Male drivers (Gd)$\:=$1 & normal operational (Vss)$\:=$1	Male drivers (Gd)$\:=$1 & absence of safety device usage (Sdu)$\:=$6
	Visibility of 200 m and more (Vis)$\:>$3 & sober or undetectable blood alcohol content (Bac)$\:>$4	Visibility of 200 m or more (Vis)$\:>$3 & driving under high alcohol content (Bac)$\:=$2	Visibility of 100–200 m or 200 m and more (Vis)$\:=$2,3 & driving under high alcohol content (Bac)$\:=$2
	Abnormal driving (Vss)$\:=$1 & absence of safety device usage (Sdu)$\:=$2	Abnormal driving (Vss)$\:=$1 & unknown safety devices (Sdu)$\:=$9	Abnormal driving (Vss)$\:=$1 & unknown or safety device usage (Sdu)$\:=$9,1
	Driving under high alcohol content (Bac)$\:=$2 & older drivers (Ag)$\:>$60	Driving under the influence of alcohol (Bac)$\:=$3 & older drivers (Ag)$\:>$50	Driving under the influence of alcohol (Bac)$\:=$1,2,3 & drivers age between 29 to 45 (Ag)$\:<$45
	Driving under low alcohol content (Bac)$\:=$1 & utilization of safety devices (Sdu)$\:=$1	Sober (Bac)$\:=$4 & utilization of safety devices (Sdu)$\:=$1	—
	Non motor vehicle involved (Mv)$\:=$0 & abnormal driving (Vss)$\:=$1	One or two vehicles involved (Mv)$\:=$1,2 & abnormal driving (Vss)$\:=$1	One or two vehicles involved (Mv)$\:=$1,2 & abnormal driving (Vss)$\:=$1

A non-parametric analysis using the Kruskal-Wallis H test was conducted to compare data across different groups (Table 10). The study focused on the effects of Bac (driving under the influence), At (type of accident), Aop (accident occurrence time), Wc (weather conditions), Ol (overloading), Vss (vehicle safety status), and Rf (road facility). Among these factors, Bac is the most significant contributor to group differences, highlighting its critical role in traffic accidents. Specifically, Vss showed the largest effect size, reflecting the substantial influence of accident type on severity levels. Similarly, Bac and At emerge as the most significant contributors to g accident severity, suggesting their pivotal role in crash severity. Notably, Bac and At exhibit substantial effects across all three levels of accident severity, demonstrating a strong and consistent association between impaired driving and adverse traffic outcomes. These findings reveal the critical importance of both accident type and driving under the influence in shaping traffic accident outcomes, emphasizing the elevated risks posed by these factors compared to others such as accident occurrence time and overloading.

**Table 10:** Non-parametric analysis: results for single factors and pairwise comparisons.

Factors	Ranking	*p*	Effect Size (η²)	Group1	Group2	Meandiff	*p*	Lower CI	Upper CI	Reject
Bac	Top3	0.0	0.1497	Fatal	Injury	0.8851	0.0	0.6252	1.145	True
				Fatal	No injury	−1.2207	0.0	−1.14818	−0.9596	True
				Injury	No injury	−2.1058	0.0	−2.2271	−1.9846	True
Aop	Top8	0.0647	0.0	Fatal	Injury	0.7619	0.0099	0.1494	1.3744	True
				Fatal	No injury	0.6693	0.0291	0.0539	1.2846	True
				Injury	No injury	−0.0926	0.7276	−0.3784	0.1931	False
Ol	Top4	0.0009	0.0016	Fatal	Injury	0.0047	0.7404	−0.0103	0.0198	False
				Fatal	No injury	0.0134	0.0948	−0.0017	0.0285	False
				Injury	No injury	0.0087	0.0109	0.0016	0.0157	True
At	Top2	0.0	0.2987	Fatal	Injury	0.9565	0.0	0.9184	0.9945	True
				Fatal	No injury	1.0327	0.0	0.9945	1.071	True
				Injury	No injury	0.0763	0.0	0.0585	0.094	True
Wc	Top5	0.0068	0.0012	Fatal	Injury	−0.0219	0.8125	−0,1055	0.0617	False
				Fatal	No injury	0.0235	0.7897	−0.0605	0.1075	False
				Injury	No injury	0.0454	0.0177	0.0063	0.0844	True
Rf	Top6	0.0114	0.0011	Fatal	Injury	0.2741	0.2143	−0.1092	0.6573	False
				Fatal	No injury	0.1575	0.6027	−0.2275	0.5426	False
				Injury	No injury	−0.1165	0.2780	−0.2953	0.0623	False
Vss	Top1	0.0	0.4454	Fatal	Injury	0.0378	0.9547	−0.268	0.3437	False
				Fatal	No injury	−0.6156	0.0	−0.9104	−0.3207	True
				Injury	No injury	−0.6534	0.0	−0.7726	−0.5342	True
Tsm	Top7	0.0383	0.0008	Fatal	Injury	0.0363	1.0	−25.1847	25.2573	False
				Fatal	No injury	3.5206	0.9432	−21.8183	28.8595	False
				Injury	No injury	3.4843	0.767	−8.2829	15.2515	False

## Conclusion

This research introduces an interpretable machine learning framework incorporating advanced models with Explainable AI (XAI) techniques to enhance prediction performance and illuminate the key factors influencing crashes and severity. Leveraging the integrated Application Platform for Public Security and Traffic Management in China from 2018 to 2021, developing and evaluating the integrated approach using two distinct datasets, we found that enhanced multi-task learning deep neural networks (Adv MT-DNN) with shared layers outperform other approaches across all severity types. Furthermore, post-hoc explanation and non-parametric analysis provide comprehensive insights into crash severity. The result reveals that accidents are usually caused by a combination of factors, with individual feature contributions and their interactions playing critical roles in shaping severity. Specifically, several key strategies to reduce accidents and severity include enforcing stricter alcohol limits, promoting safety device usage, enhancing driver training, particularly for younger and older drivers, and providing weather alerts and alternative routes to mitigate risks associated with adverse weather. Additionally, interventions targeting specific demographic groups, such as promoting safety awareness among drivers with lower education levels, could help reduce the risk of fatal accidents.

Several limitations warrant consideration. The exclusive reliance on officially reported incidents introduces potential selection bias, particularly regarding unreported minor crashes. Although our sampling approach addresses class imbalance, residual challenges persist in data heterogeneity and outcome consistency across regions. Future research should consider integrating various data sources, such as vehicle traffic volume and video data, to enhance the understanding of factors that influence crash severity. Concurrently, incorporating high-resolution road geometry measurements and establishing cross-regional validation protocols through federated learning architectures would strengthen the model’s geographical generalizability, particularly for China’s diverse transportation ecosystems.

## Data Availability

Data will be available on request. The RTA dataset is collected from Addis Ababa Sub city police departments for Masters research work, doi: 10.17632/xytv86278f.1.
